# Survey of pharmacy strategic planning and execution in US health systems

**DOI:** 10.1093/ajhp/zxag060

**Published:** 2026-03-04

**Authors:** John S Clark, Tara Behring Vlasimsky, Toni Fera, Barbara B Nussbaum

**Affiliations:** Michigan Medicine, Ann Arbor, MI, USA; Intermountain Health, Denver, CO, USA; Contractor, Greater Pittsburgh Area, PA, USA; ASHP Research and Education Foundation, Bethesda, MD, USA

**Keywords:** health-system pharmacy, leadership, strategic planning, US health systems

## Abstract

**Purpose:**

Results of a survey on the state of enterprise-wide pharmacy strategic planning and execution in US health systems are presented.

**Methods:**

An online survey instrument was developed based on guidance by an expert advisory committee. The survey covered 5 domains: (1) current state of strategic planning, (2) plan-development process, (3) stakeholder engagement, (4) plan dissemination, and (5) demographics.

**Results:**

Of the 1,092 health-system pharmacy practice leaders surveyed, 225 (21%) responded. Among the respondents, 187 (83%) had a current enterprise-wide pharmacy strategic plan (EWPSP) or were slated to develop one in the next 12 months. The 165 respondents actively engaged with an EWPSP responded to questions about their process. Most EWPSPs covered a timeframe of 3 to 5 years and were revisited at least annually. The strategic plans of more than two-thirds of respondents included (1) goals; (2) actions to achieve each goal; (3) mission, vision, and values of the pharmacy enterprise; and (4) the accountable person or team for each goal. Most respondents agreed strongly that their plan helped showcase pharmacy initiatives to external stakeholders and facilitated networking across the pharmacy enterprise. Respondents indicated it would be helpful to have additional resources to identify emerging trends, measure plan performance, and align plans with service line priorities. Based on the survey results and their own experience, the authors offer advice on ways to enhance the effectiveness of enterprise-wide pharmacy strategic planning.

**Conclusion:**

EWPSPs in US health systems are developed and executed using a variety of resources and methods and are aligned with the overall goals of their organizations. Survey respondents indicated that their strategic plans help (1) expand existing services or develop new services and (2) gain support from internal and external stakeholders.

The importance of strategic planning in leading the pharmacy enterprise in health systems has been well established.^1-5^ However, there have been only a few reports on the prevalence and nature of strategic planning in health-system pharmacy. In a survey of pharmacy directors in the late 1980s, 79% of respondents indicated they had specific written documented goals and objectives for the department.^6^ A survey of “institutional pharmacists” more than 3 decades ago reported 63% of pharmacy directors had a strategic plan.^7^ In an unpublished ASHP survey of pharmacy directors in 2014, only 49% reported having a pharmacy strategic plan (C. Bush, D. Chen, W.A. Zellmer, ASHP, email, October 21, 2024). A few published reports describe service line– or team-specific strategic planning in health-system pharmacy.^8-10^ No reports were found that described tools and resources health-system pharmacists use in developing, implementing, and assessing their strategic plans.

The objective of this survey, conducted under the auspices of the ASHP Foundation, was to document the current state of strategic planning in US health-system pharmacy. Information was collected on many facets of pharmacy strategic planning processes, including reasons for engaging in the process, top-priority issues covered in current plans, tools and resources used in planning, and assessment of outcomes of the process.

## Methods

### Survey design

The ASHP Foundation appointed an advisory committee to design and pilot test the survey (see sidebar). The committee was comprised of health-system pharmacists with strategic planning experience, representing various types of practice settings and regions of the country.

Advisory Committee **Lindsey Amerine**, PharmD, MS, BCPS, CPEL, FASHP

*Cleveland Clinic, Cleveland, OH*

**John S. Clark**, PharmD, MS, BCPS, FASHP

*Michigan Medicine, Ann Arbor, MI*

**James M. Hoffman,** PharmD, MS, FASHP

*St. Jude Children’s Research Hospital, Memphis, TN*

**Thomas J. Johnson,** PharmD, MBA, BCCP, BCPS, FACHE, CPEL

*Avera Health, Sioux Falls, SD*

**Trisha Jordan**, PharmD, MS

*The Ohio State University Wexner Medical Center and The Ohio State University College of Pharmacy, Columbus, OH*

**Mary Beth Lang**, ScD, MSPPM, RPh

*Kaiser Permanente, Oakland, CA*

**Jennifer Miles**, PharmD, BCACP

*Jackson Health System, Miami, FL*

**Michael Nnadi,** PharmD, MHS, BSP

*Harris Health System, Greater Houston, TX*

**Kuldip Patel**, PharmD

*Duke University Hospital, Durham, NC*

**Michael Sanborn,** RPh, MS, FASHP, FACE

*Baylor Scott & White Health, Dallas, Tx*

**Deb Simonson**, PharmD, CPEL

*Ochsner Health, New Orleans, LA*

**Mark Sullivan,** PharmD, MBA, BCPS

*Vanderbilt University Medical Center, Nashville, TN*

**Tara Behring Vlasimsky**, PharmD, MBA, BCACP, FASHP

*Intermountain Health, Denver, CO*

**Special Advisors**

**Marianne F. Ivey,** PharmD, MPH, FASHP, FFIP

*University of Cincinnati Pharmacy Practice and Administrative Sciences, Kenmore (Seattle), WA*

**William Zellmer**, BSPharm, MPH

*Pharmacy Foresight Consulting, Bethesda, MD*

**David Zilz**, MS, FASHP

*UWHealth and University of Wisconsin School of Pharmacy (Retired), Iola, WI*

**Staff**

**Barbara B. Nussbaum**, BSPharm, PhD

*ASHP Research and Education Foundation, Bethesda, MD*

**David Chen,** BSPharm, MBA

*American Society of Health-System Pharmacists, Bethesda, MD*

**Cherrelle Draughan**, BA, MS

*ASHP Research and Education Foundation, Bethesda, MD*

**Toni Fera,** BSPharm, PharmD

*Independent Consultant (Contractor), Pittsburgh, PA*

**Douglas J. Scheckelhoff**, MS, FASHP

*American Society of Health-System Pharmacists, Bethesda, MD*

The Checklist for Reporting Results of Internet E-Surveys (CHERRIES)^11^ was applied to survey development, pretesting, administration, and reporting of results, and the research was conducted in accordance with the principles of the Declaration of Helsinki.^12^

In the absence of a standardized questionnaire to address the research objectives, a 28-question electronic survey instrument was developed, refined, and tested with input from the advisory committee to assess 5 domains: The current state, strategic-plan development process, stakeholder engagement, plan dissemination, and demographics. Both published and unpublished literature on strategic planning were also reviewed to identify relevant content and further inform the development of questions within the target domain.^6,7^ Of the survey’s 28 questions, 19 were multiple-select items. Three of the multiple-choice questions were used to identify eligible survey participants (ie, practitioners who had a strategic plan, intended to develop a strategic plan, or were otherwise involved in strategic planning). Nine matrix-style Likert scale questions were also used. The survey provided the following definition of an “enterprise-wide pharmacy strategic plan” (EWPSP):

A plan that encompasses the overall pharmacy enterprise in an institution or health system. The plan may reflect a pharmacy enterprise in a single hospital or the overarching pharmacy enterprise of a multihospital system. It may be a standalone plan or be integrated within an organization-wide strategic plan.

Some questions were designated as mandatory and were required to be answered. Partial responses were included in the analysis. After the advisory committee completed its work in crafting the online survey, the instrument was tested by 10 pharmacy leaders for additional refinement.

### Survey sample and administration

A sampling frame of 4,926 general and children’s medical-surgical hospitals in the United States was constructed from the IQVIA database.^13^ Federal hospitals were excluded. Hospitals were stratified by bed size before sampling to reflect the diversity of hospital sizes across the population. Through random selection, the final sample of 1,274 hospitals was established with similar bed-size sampling as in the 2023 ASHP National Survey of Pharmacy Practice in Hospital Settings.^14^ Internal ASHP databases were used to update director of pharmacy contacts from the IQVIA list.

Estimating that about 50% of the sample would have a strategic plan (C. Bush, D. Chen, W.A. Zellmer, ASHP, email, October 21, 2024), it was calculated that 95 to 357 respondents would be needed to achieve a margin of error of ±10% to ±5% at a 95% confidence level.^15^

The survey was administered online using Qualtrics (Qualtrics, Provo, UT) between April 3 and June 1, 2025. The individuals surveyed were advised that respondents would be eligible to opt into a random drawing to receive 1 of 6 prizes (a conference registration or products valued at $795). Emails not deliverable reduced the sample size to 1,092.

Panelists were contacted up to 8 times during the survey period. No personal information was collected and stored with survey responses. Respondents were invited to provide contact information for follow-up work.

## Results

### Respondent characteristics

**Figure 1.**
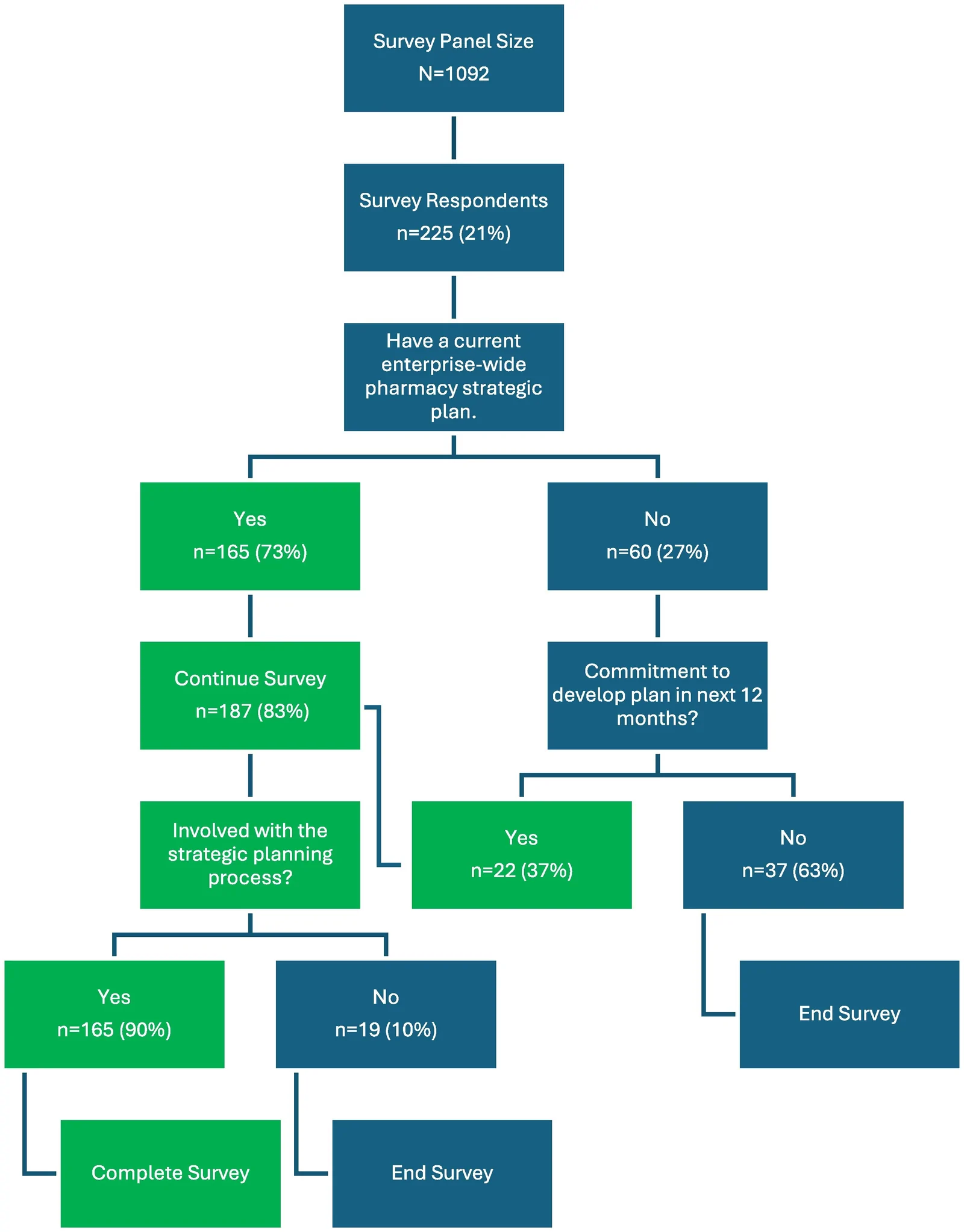
Screening algorithm for survey panelists.

Of the 1,092 panelists surveyed, 225 (21%) responded; 187 (83% of respondents) indicated they either had a current EWPSP or there was a commitment to develop one in the next 12 months. Within this cohort of 187, only the 165 who indicated they were involved in the EWPSP process (as primary leaders or participants) were invited to complete the balance of the survey (Figure 1). The EWPSP results in this report represent these 165 respondents. Table 1 shows demographic data on the whole group of 225 respondents. Participants were dispersed broadly across regions.

**Table 1.** Demographics of Survey Respondents

Characteristic	No. (%) responding
Region (N = 225)	
Midwest	82 (36)
South	63 (28)
Northeast	48 (21)
West	32 (14)
Staffed beds at primary practice setting (N = 225)	
0-49	43 (19)
50-199	26 (12)
200-299	30 (13)
300-399	41 (18)
400-599	43 (19)
600+	42 (19)
Primary work setting (n = 170)	
Community hospital	72 (42)
Academic medical center	51 (30)
Corporate/system level	26 (15)
Critical access hospital	12 (7)
Children’s hospital	5 (3)
Centralized shared services	1 (1)
Community/retail pharmacy	1 (1)
Home infusion/home care	1 (1)
Other	1 (1)
Work in a multihospital health system (n = 170)	
Yes	152 (89)
No	18 (11)
Engagement in the enterprise-wide pharmacy strategic planning process (n = 184)	
Among the primary leaders of the process	109 (59)
Participant in the process	56 (30)
Not involved in the process	19 (10)

The most frequent reasons given for not having or not planning to have an EWPSP (37 respondents) were lack of a perceived priority within the pharmacy enterprise, a change in organizational leadership, or a lack of “know-how.”

### Nature of current strategic plan

Most respondents (53%) indicated their EWPSP covered a timeframe of 3 to 5 years, followed by 36% indicating less than 3 years. (The remaining responses were scattered among other timeframes.)

**Figure 2.**
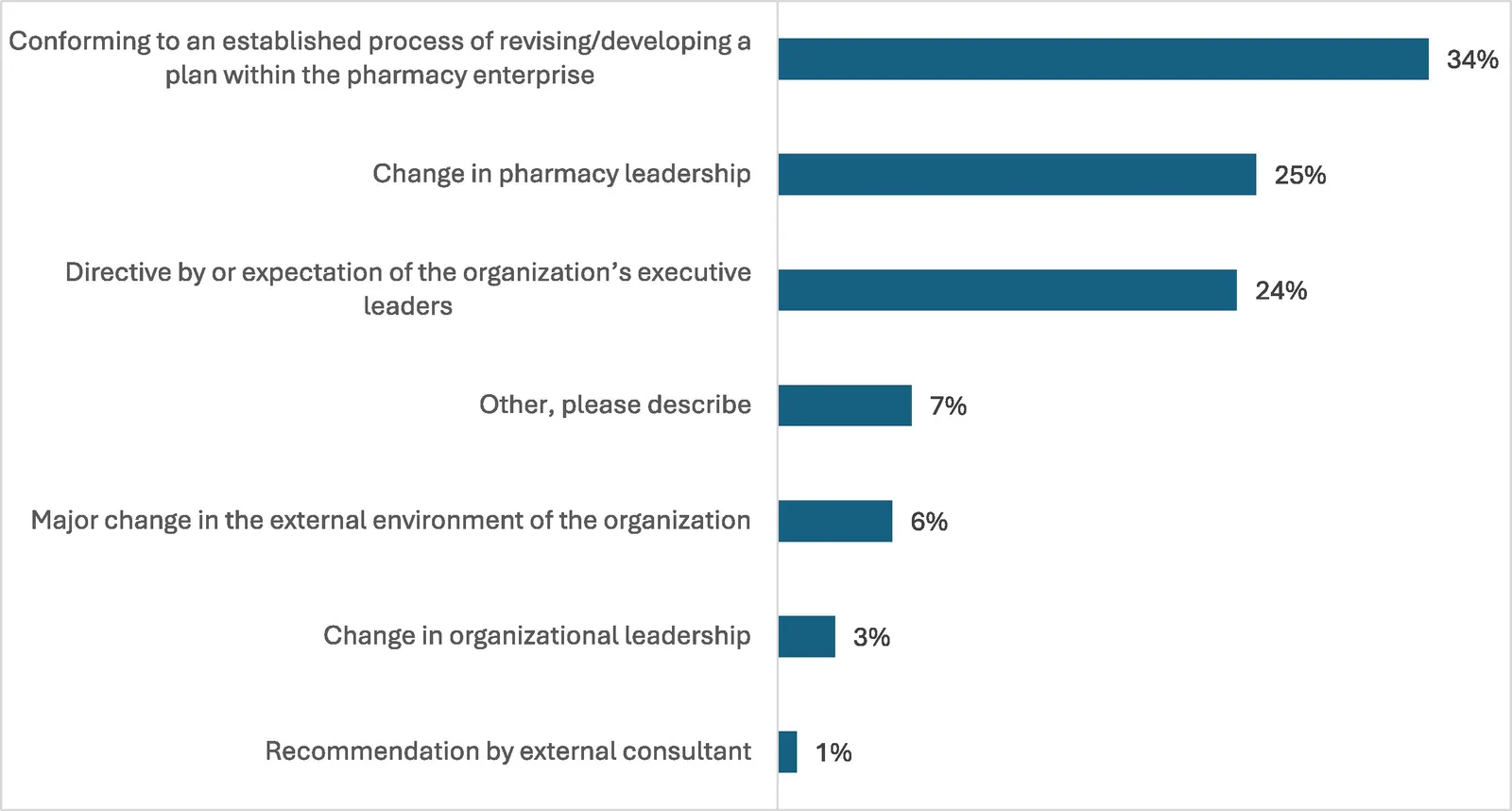
Primary driver for initiating the most recent enterprise-wide pharmacy strategic plan (n = 126).

**Figure 3.**
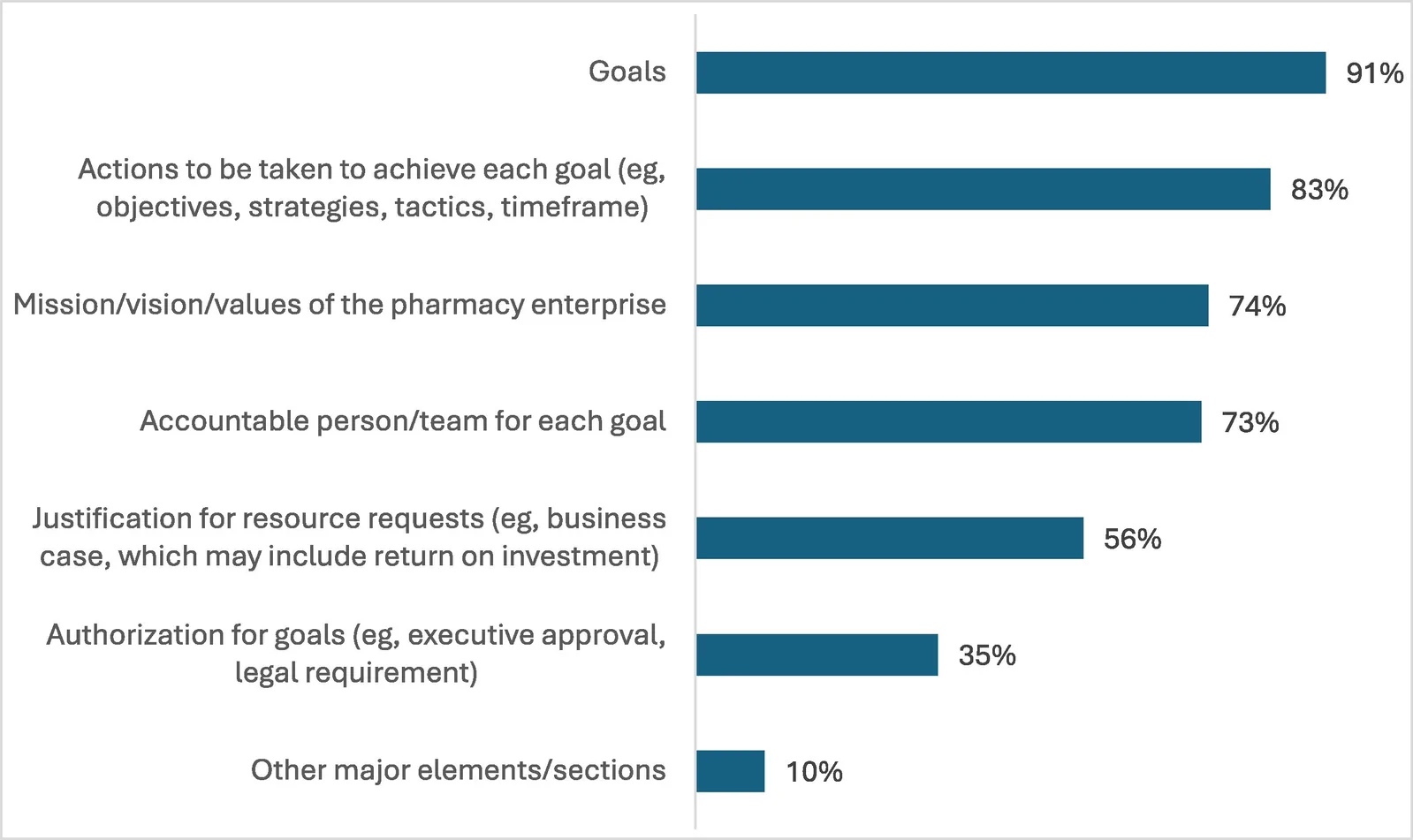
Elements included in the enterprise-wide strategic plan (n = 150).

The most common reasons for initiating the current plan included (1) conformance with an established planning process (34%); (2) a change in pharmacy leadership (25%); or (3) a directive or expectation of organization leadership (24%) (Figure 2). Over two-thirds of respondents reported that their strategic plans included (Figure 3):

- Goals;
- Actions to be taken to achieve each goal (eg, objectives, strategies, tactics, timeframe);
- Mission, vision, and values of the pharmacy enterprise; and
- The accountable person or team for each goal.

**Figure 4.**
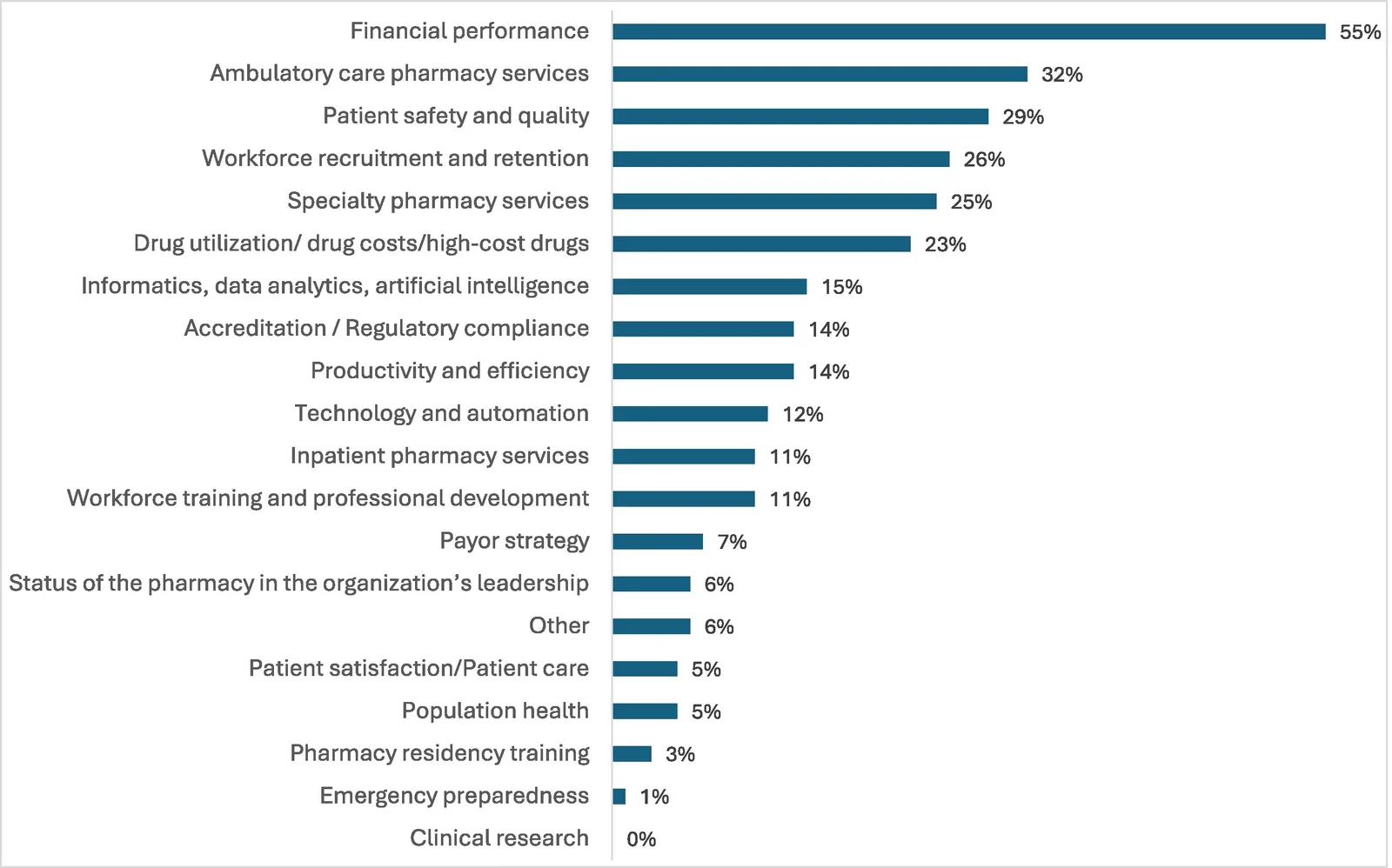
Top 3 strategic priorities in current plan (n = 123).

Respondents indicated that the financial performance, ambulatory care pharmacy services, and patient safety and quality areas were among the top 3 strategic priorities in their plans (listed in rank order) (Figure 4).

A large majority of respondents (81%) indicated that 1 or more pharmacy team members have direct involvement in developing and implementing the organization-wide strategic plan.

### Strategic plan development

Respondents were asked to rate the relative frequency with which various “factors, assessments, or activities” were included or considered in cycles of the planning process, using a 5-point Likert scale (never, rarely, sometimes, often, always) (Table 2). Cited as most frequently included/considered, in rank order by mean, were:

1. Pharmacy plan alignment with the organization mission/vision/goals
2. Expressing a vision for the future of the pharmacy enterprise
3. Analysis of the current state
4. Assigning priorities among pharmacy’s strategic goals
5. Identifying relevant future trends
6. Pharmacy plan alignment across organization service lines

**Table 2.** Extent to Which Factors, Assessment, or Activities are Included or Considered in the Enterprise-Wide Pharmacy Strategic Planning Process

Item	No. (%) responding					Mean ranking^a^
	Never	Rarely	Sometimes	Often	Always	
Pharmacy plan alignment with the organization mission/vision/goals (n = 134)	1 (1)	3 (2)	8 (6)	29 (22)	93 (69)	4.57
Expressing a vision for the future of the pharmacy enterprise (n = 134)	1 (1)	3 (2)	11 (8)	43 (32)	76 (57)	4.42
Analysis of the current state (n = 134)	0	5 (4)	18 (13)	37 (28)	74 (55)	4.34
Assigning priorities among pharmacy's strategic goals (n = 129)	0	4 (3)	9 (7)	56 (43)	60 (47)	4.33
Identifying relevant future trends (n = 134)	0	1 (1)	22 (16)	52 (39)	59 (44)	4.26
Pharmacy plan alignment across organization service lines (n = 134)	0	5 (4)	21 (16)	43 (32)	65 (49)	4.25
Engaging relevant stakeholders in developing parts of the pharmacy plan that affects them (n = 129)	1 (1)	4 (3)	21 (16)	46 (36)	57 (44)	4.19
Capital and operating resource requirements (n = 129)	4 (3)	3 (2)	17 (13)	47 (36)	58 (45)	4.18
Fostering understanding of the plan among key stakeholders (n = 129)	2 (2)	4 (3)	19 (15)	48 (37)	56 (43)	4.18
Future staffing needs (pharmacists and technicians) (n = 130)	2 (2)	7 (5)	20 (15)	50 (38)	51 (39)	4.08
Scheduled assessment of progress in achieving the plan’s goals (n = 129)	0	10 (8)	20 (16)	49 (38)	50 (39)	4.08
The extent to which pharmacy’s plan can influence the organization-wide strategic plan (n = 134)	2 (1)	7 (5)	34 (25)	48 (36)	43 (32)	3.92
Internal benchmarking (e.g., drug utilization trends, productivity targets) (n = 132)	3 (2)	9 (7)	25 (19)	53 (40)	42 (32)	3.92
Aspirational external recommendations for advancing the profession of pharmacy (e.g., ASHP Practice Advancement Initiative) (n = 134)	8 (6)	10 (7)	29 (22)	52 (39)	35 (26)	3.72
Succession planning (n = 130)	7 (5)	21 (16)	46 (35)	37 (28)	19 (15)	3.31
External benchmarking (e.g., productivity and staffing databases) (n = 134)	7 (5)	24 (18)	47 (35)	36 (27)	20 (15)	3.28
Comparison of the pharmacy enterprise to competing organizations (n = 134)	12 (9)	26 (19)	45 (34)	34 (25)	17 (13)	3.13
Recommendations of external consultants (n = 130)	14 (11)	42 (32)	54 (42)	17 (13)	3 (2)	2.64
Requirements for becoming an ASHP-certified Center of Excellence (n = 130)	45 (35)	33 (25)	25 (19)	22 (17)	5 (4)	2.3

^a^Each item was rated with a 5-point Likert scale ranging from 1 = “never” to 5 = “always”

**Figure 5.**
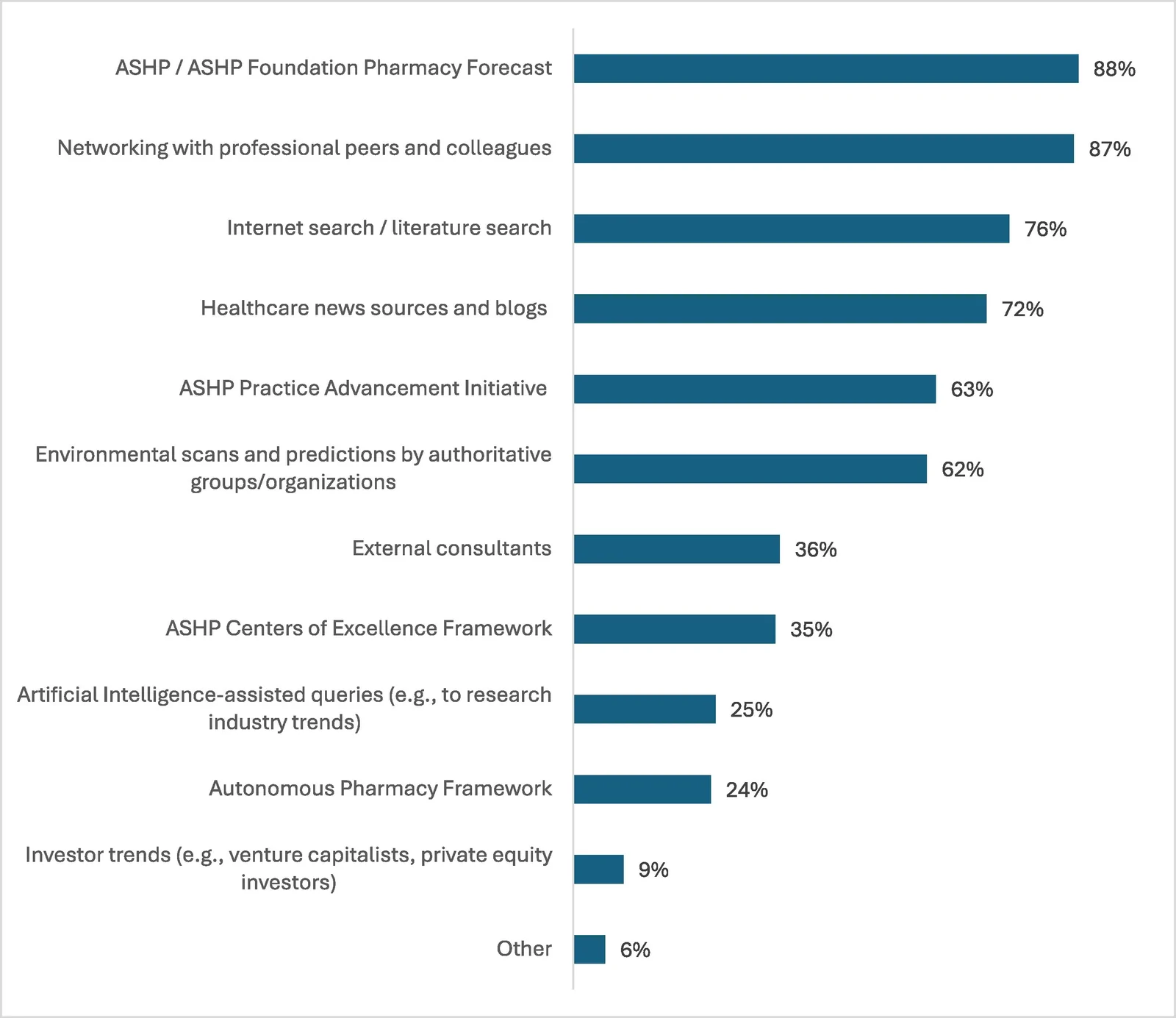
External resources used to conduct an environmental scan (n = 125).

More than 70% of respondents indicated they used the following external sources to identify trends that might have a bearing on the pharmacy enterprise (ie, in environmental scanning) (listed in rank order) (Figure 5):

1. The ASHP/ASHP Foundation Pharmacy Forecast (88%)
2. Networking with professional peers and colleagues (87%)
3. Internet/literature search (76%)
4. Healthcare news sources and blogs (72%)

**Figure 6.**
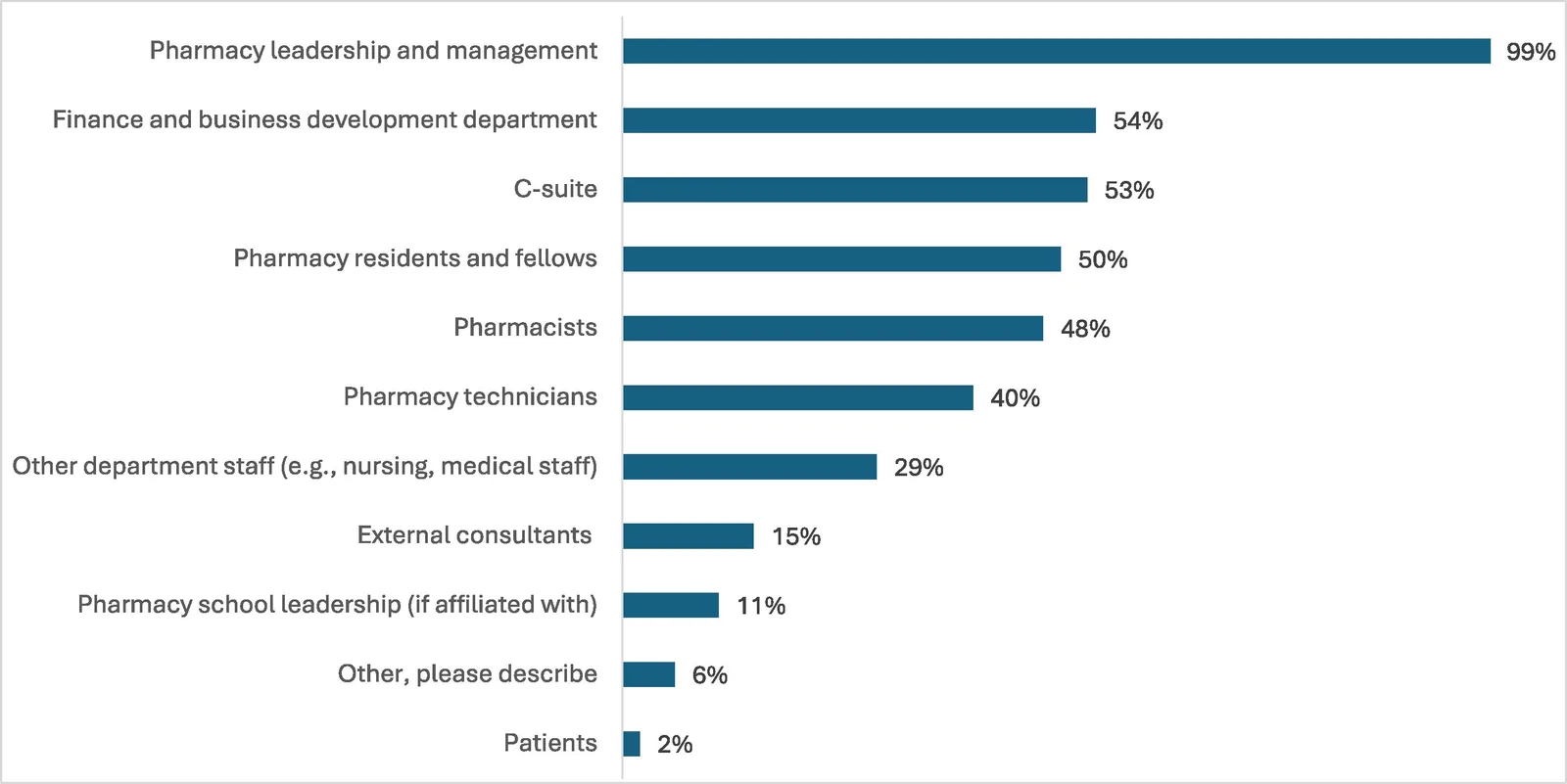
Members of the core team directly involved in building the enterprise-wide pharmacy strategic plan (n = 126).

**Figure 7.**
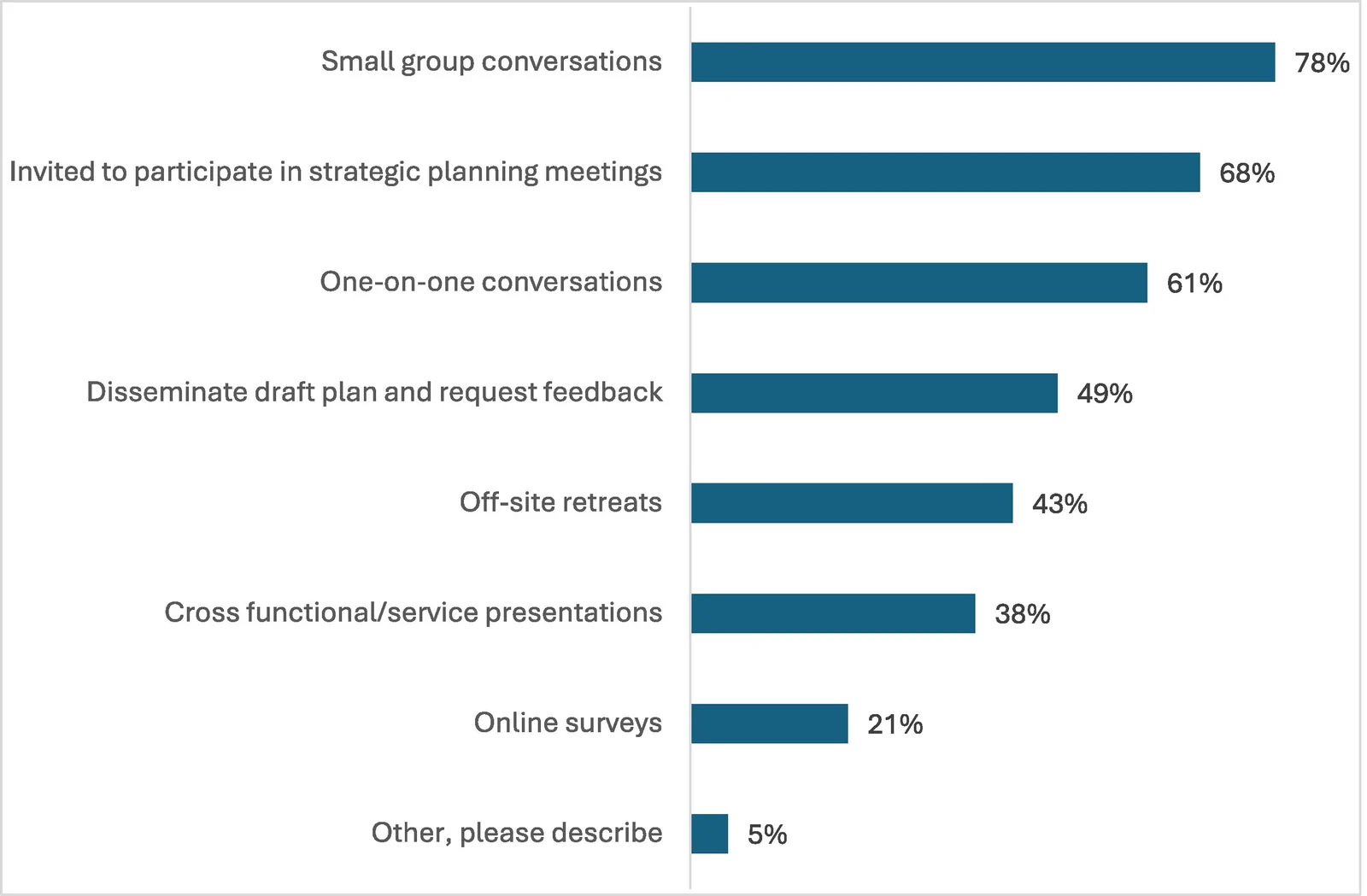
How stakeholders are actively engaged in the strategic planning process (n = 126).

Respondents reported that “pharmacy leadership and management” was almost always directly involved in building the EWPSP (Figure 6). Figure 7 reports the frequency with which respondents used various techniques to engage stakeholders in the planning process.

When asked to assess the overall value of their EWPSP, respondents strongly agreed that their plan helped improve pharmacy operations and helped advance the pharmacy profession (Table 3). Respondents had somewhat less favorable assessments of the following facets of their planning process: (1) alignment across pharmacy service lines, (2) balance between near-term operational issues and emerging trends, (3) timeliness of plan implementation, and (4) effectiveness of setting priorities.

**Table 3.** Overall Value of the Enterprise-Wide Pharmacy Strategic Plan

Item	No. (%) responding					Mean ranking^a^
	Strongly disagree	Disagree	Neutral	Agree	Strongly agree	
Having a current strategic plan is critical to improving pharmacy operations. (n = 116)	0	0	7 (6)	34 (29)	75 (65)	4.59
Execution of the plan helps to advance the profession of pharmacy. (n = 116)	0	0	10 (9)	44 (38)	62 (53)	4.45
Overall, the strategic plan is well aligned across service lines within the pharmacy enterprise. (n = 116)	1 (1)	4 (3)	10 (9)	68 (59)	33 (28)	4.10
The focus of the plan has an appropriate balance between near-term operational issues and long-term emerging trends. (n = 116)	2 (2)	4 (3)	8 (7)	69 (59)	33 (28)	4.09
Execution of the plan occurs within the defined timeframe. (n = 116)	1 (1)	6 (5)	24 (41)	63 (54)	22 (19)	3.85
There is an effective process for setting priorities for plan initiatives. (n = 116)	1 (1)	7 (6)	23 (20)	62 (53)	23 (20)	3.85

^a^Each item was rated with a 5-point Likert scale ranging from 1 = “strongly disagree” to 5 = “strongly agree”

### Plan dissemination and implementation

Task assignments in implementing a strategic plan, and a mechanism to monitor plan progress (eg, dashboards), were “often” or “always” integrated into monitoring the execution of the EWPSP (79% and 74%, respectively) (Table 4). Other frequent facets of plan execution included (1) regular communication of plan progress to stakeholders within the pharmacy enterprise, (2) revisiting the plan at regular intervals and adjusting as needed, (3) business plans, and (4) regular communication of plan progress to stakeholders external to the pharmacy enterprise.

**Table 4.** Extent Elements are Integrated Into Pharmacy Strategic Plan Execution

Item	No. (%) responding					
	Never	Rarely	Sometimes	Often	Always	Mean ranking^a^
Assignment of tasks (n = 120)	1 (1)	6 (5)	18 (15)	43 (36)	52 (43)	4.16
Mechanism to monitor plan progress and performance for defined goals, strategies, tactics (eg, dashboard or other reporting tool) (n = 120)	1 (1)	6 (5)	25 (21)	45 (38)	43 (36)	4.03
Regular communication of plan progress to stakeholders within the pharmacy enterprise (n = 120)	0	6 (5)	29 (24)	53 (44)	32 (27)	3.92
Revisiting plan at regular intervals and adjust as needed (n = 120)	2 (2)	7 (6)	31 (26)	43 (36)	37 (31)	3.88
Business plan(s) (eg, financial and staffing resources to support initiatives, return on investment projections) (n = 120)	3 (3)	8 (7)	32 (27)	49 (41)	28 (23)	3.76
Regular communication of plan progress to stakeholders, external to the pharmacy enterprise (n = 120)	3 (3)	9 (8)	39 (33)	49 (41)	20 (17)	3.62

^a^Each item was rated with a 5-point Likert scale ranging from 1 = “never” to 5 = “always”

Respondents were asked to assess the outcomes from their most recent planning cycle (Table 5). Two-thirds indicated that goals in the current plan were met or exceeded within the designated timeframe “often” or “always.” A large majority of respondents (83%) indicated that elimination or reduction in pharmacy services “rarely” or “never” was an outcome of the most recent planning cycle, whereas 91% of respondents indicated that, at least sometimes, EWPSP implementation resulted in establishing new or expanded pharmacy services.

**Table 5.** Implementation Outcomes of the Most Recent Enterprise-Wide Pharmacy Strategic Planning Cycle

Item	No. (%) responding					Mean ranking^a^
	Never	Rarely	Sometimes	Often	Always	
Goals in the current plan were met or exceeded within the designated timeframe (n = 119)	1 (1)	3 (3)	35 (29)	70 (59)	10 (8)	3.71
Established new or expanded pharmacy services (n = 119)	2 (2)	9 (8)	30 (25)	59 (50)	19 (16)	3.71
Reprioritization of current goals or shift in focus to new strategic priorities (n = 119)	0	16 (13)	44 (37)	47 (39)	12 (10)	3.46
Process changes were made in the health system’s medication-use system (including medication administration) (n = 118)	3 (3)	19 (16)	52 (44)	33 (28)	11 (9)	3.25
Additional financial resources were allocated to pharmacy (eg, capital or operating dollars) (n = 119)	5 (4)	23 (19)	41 (34)	37 (31)	13 (11)	3.25
Approval of additional pharmacy FTEs (n = 119)	6 (5)	22 (18)	51 (43)	34 (29)	6 (5)	3.10
Redeployment of pharmacy FTEs (n = 118)	7 (6)	19 (16)	58 (49)	29 (25)	5 (4)	3.05
Reduction of financial resources allocated to pharmacy (eg, capital or operating dollars) (n = 119)	19 (16)	51 (43)	34 (29)	9 (8)	6 (5)	2.43
Reduction of pharmacy FTEs (n = 119)	41 (34)	47 (39)	24 (20)	4 (3)	3 (3)	2.00
Elimination or reduction of pharmacy services (n = 119)	47 (39)	52 (44)	16 (13)	3 (3)	1 (1)	1.82

Abbreviation: FTE, full-time equivalent.

^a^Each item was rated with a 5-point Likert scale ranging from 1= “never” to 5 = “always”

As shown in Table 6, respondents reported that progress toward achievement of EWPSP goals was frequently used in:

1. Establishing performance objectives for leaders/managers in the pharmacy enterprise
2. Reporting the status of plan execution to executive leaders
3. Creating action plans for pharmacy service areas
4. Monitoring by designated persons for each goal in the plan
5. Creating agendas for staff meetings
6. Establishing performance objectives for pharmacy staff members (other than leadership)
7. Evaluating progress by a formal pharmacy committee

**Table 6.** Monitoring Progress Toward Achieving the Goals of the Enterprise-Wide Pharmacy Strategic Plan

Item	No. (%) responding					Mean ranking^a^
	Never	Rarely	Sometimes	Often	Always	
Establishing performance objectives for leaders/managers in the pharmacy enterprise (n = 116)	2 (2)	6 (5)	23 (20)	56 (48)	29 (25)	3.90
Reporting status of plan implementation to executive leaders (n = 116)	3 (3)	11 (9)	33 (28)	38 (33)	31 (27)	3.72
Creating action plans for pharmacy service areas (n = 116)	4 (3)	6 (5)	33 (28)	52 (45)	21 (18)	3.69
Monitoring by designated persons for each goal in the plan (n = 116)	3 (3)	15 (13)	31 (27)	39 (34)	28 (24)	3.64
Creating agendas for staff meetings (n = 115)	6 (5)	16 (14)	33 (29)	34 (30)	26 (23)	3.50
Establishing performance objectives for pharmacy staff members (other than leadership) (n = 116)	5 (4)	15 (13)	37 (32)	42 (36)	17 (15)	3.44
Evaluating progress by a formal pharmacy committee (n = 116)	9 (8)	19 (16)	28 (24)	38 (33)	22 (19)	3.39
Evaluating progress by a formal non-pharmacy committee (n = 116)	22 (19)	33 (28)	37 (32)	18 (16)	6 (5)	2.59
Allocation of financial bonuses to pharmacy leaders/managers (n = 116)	45 (39)	16 (14)	26 (22)	19 (16)	10 (9)	2.42
Allocation of financial bonuses to pharmacy staff members (other than leadership) (n = 116)	78 (67)	15 (13)	13 (11)	5 (4)	5 (4)	1.66

^a^Each item was rated with a 5-point Likert scale ranging from 1= “never” to 5 = “always

Financial bonuses linked to achieving strategic goals were rarely used.

**Figure 8.**
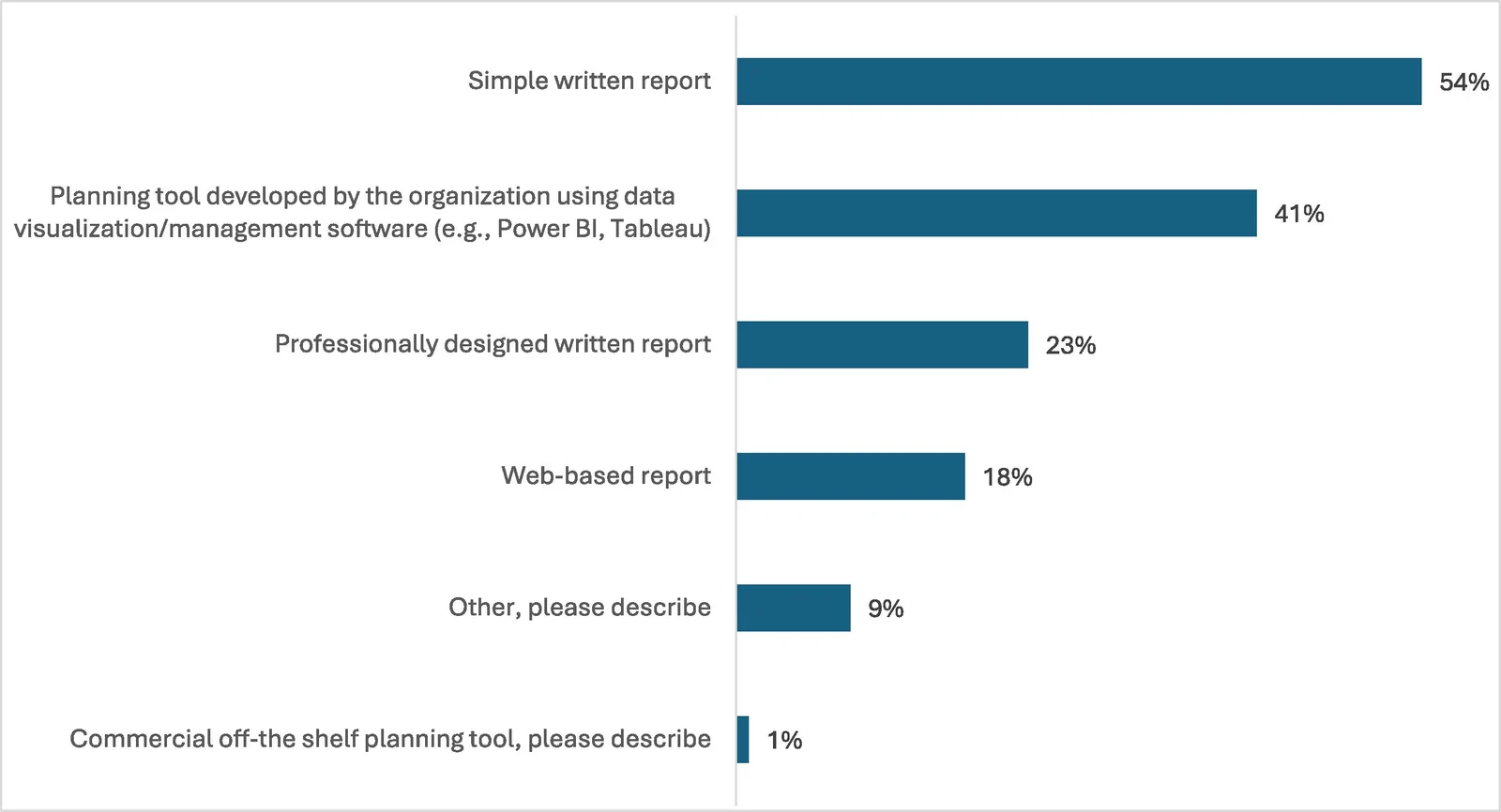
Format for dissemination of enterprise-wide pharmacy strategic plan (n = 117).

Plans were most often disseminated as a simple written report (54%) or via a planning tool developed by the organization using data visualization/management software (41%) (Figure 8).

### Plan performance review and refinement

**Figure 9.**
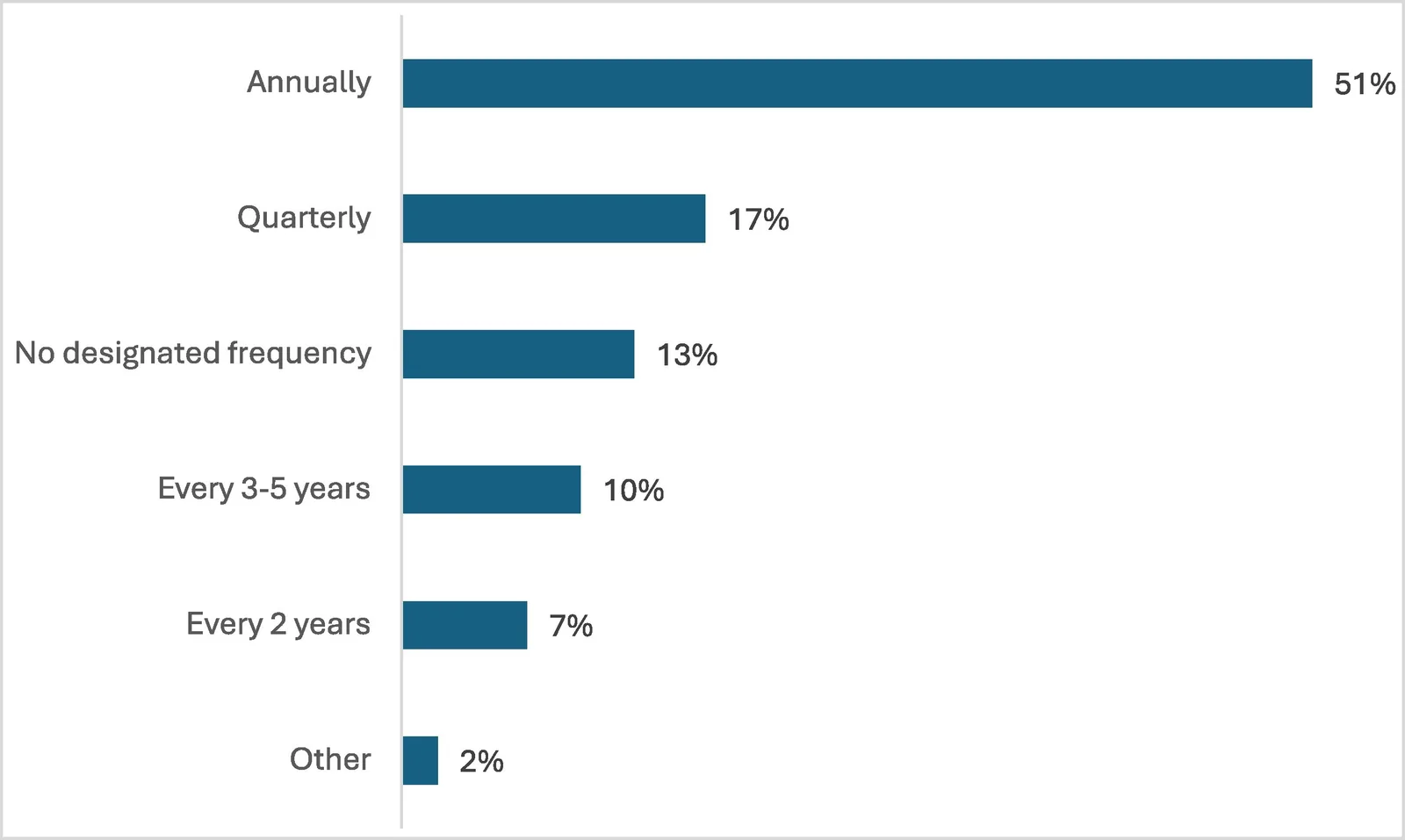
Frequency of formal review and updating of enterprise-wide pharmacy strategic plan (n = 125).

Respondents indicated that they formally review and update their overall EWPSP annually (51%) or quarterly (17%) (Figure 9). Some respondents (13%) indicated that they did not have a designated frequency for review of their planning.

Respondents were asked how frequently certain situations have caused a major re-examination in the EWPSP. As Table 7 shows, the most likely to trigger such action were (1) major changes in the external environment; (2) legislative, regulatory, or accreditation changes; and (3) unanticipated resource restrictions. Among the choices in this survey item, changes in organization or pharmacy leadership were least likely to cause a review of the plan.

**Table 7.** Situations Causing Re-Examination or Adjustment in the Enterprise-Wide Pharmacy Strategic Plan

Item	No. (%) responding					Mean ranking^a^
	Never	Rarely	Sometimes	Often	Always	
Major changes in the external environment (n = 124)	2 (2)	11 (9)	52 (42)	44 (35)	15 (12)	3.48
Legislative, regulatory, or accreditation changes (n = 123)	2 (2)	16 (13)	60 (49)	37 (30)	8 (7)	3.27
Unanticipated resource restrictions (n = 123)	4 (3)	15 (12)	57 (46)	39 (32)	8 (7)	3.26
Target dates for implementation became unrealistic (n = 123)	4 (3)	20 (16)	65 (53)	28 (23)	6 (5)	3.10
Decline in organizational revenue (n = 122)	11 (9)	30 (25)	52 (43)	22 (18)	7 (6)	2.87
Change in organization leadership (n = 122)	6 (5)	47 (39)	44 (36)	18 (15)	7 (6)	2.78
Change in pharmacy leadership (n = 124)	12 (10)	42 (34)	46 (37)	15 (12)	9 (7)	2.73

^a^Each item was rated with a 5-point Likert scale ranging from 1= “never” to 5 = “always”

When asked about the benefits of their EWPSP, most respondents agreed strongly that their plan showcased pharmacy initiatives to external stakeholders (Table 8). Other highly rated benefits included (1) facilitating networking across the pharmacy enterprise, (2) showcasing pharmacy’s contribution to the organization’s overall strategic plan, and (3) creating accountability for implementing priorities.

**Table 8.** Benefits of an Enterprise-Wide Pharmacy Strategic Plan

Item	No. (%) responding					Mean ranking^a^
	Strongly disagree	Disagree	Neutral	Agree	Strongly agree	
Showcase pharmacy initiatives to external stakeholders (n = 116)	1 (1)	2 (2)	12 (10)	40 (34)	61 (53)	4.36
Facilitate networking across the pharmacy enterprise (n = 116)	1 (1)	2 (2)	10 (9)	47 (41)	56 (48)	4.34
Showcase pharmacy contribution to overall organization strategic plan (n = 116)	2 (2)	4 (3)	11 (9)	45 (39)	54 (47)	4.25
Facilitate team building (n = 116)	1 (1)	2 (2)	14 (12)	51 (44)	48 (41)	4.23
Create accountability for execution (n = 116)	1 (1)	2 (2)	15 (13)	51 (44)	47 (41)	4.22
Gain organizational support for priorities (n = 116)	2 (2)	4 (3)	14 (12)	54 (47)	42 (36)	4.12
Assist with prioritizing resource allocation (n = 116)	3 (3)	3 (3)	14 (12)	58 (50)	38 (33)	4.08
Drive broader organizational initiatives (n = 116)	2 (2)	8 (7)	18 (16)	45 (39)	43 (37)	4.03
Facilitate networking across the organization (n = 116)	1 (1)	2 (2)	24 (21)	54 (47)	35 (30)	4.03
Gain buy-in from external stakeholders for new pharmacy initiatives (n = 116)	2 (2)	4 (3)	23 (20)	51 (44)	36 (31)	3.99
Identify issues to inform the organization’s overall strategic plan (n = 116)	3 (3)	7 (6)	18 (16)	49 (42)	39 (34)	3.98
Gain pharmacy staff buy-in for new initiatives (n = 116)	3 (3)	6 (5)	20 (17)	56 (48)	31 (27)	3.91
Develop mid-manager leadership skills (n = 116)	3 (3)	4 (3)	23 (20)	57 (49)	28 (24)	3.87

^a^Each item was rated with a 5-point Likert scale ranging from 1= “strongly disagree” to 5 = “strongly agree”

### New resources that would be helpful

Respondents strongly agreed that additional tools and resources in the following areas of the planning process would be helpful: (1) identifying emerging trends likely to have a future impact on the pharmacy enterprise, (2) measuring and monitoring plan performance, and (3) aligning plans with service line priorities (Table 9).

**Table 9.** Additional Education, Tools, and Resources to Support Strategic Planning

Item	No. (%) responding					Mean ranking^a^
	Strongly disagree	Disagree	Neutral	Agree	Strongly agree	
Identifying emerging trends that are likely to have a future impact on the pharmacy enterprise (n = 116)	0	1 (1)	4 (3)	44 (38)	67 (58)	4.53
Measuring and monitoring performance (e.g., productivity; patient safety; staff wellness; drug use trends) (n = 116)	0	5 (4)	12 (10)	32 (28)	67 (58)	4.39
Aligning plans for the pharmacy enterprise with organizational or service line priorities (n = 116)	0	1 (1)	13 (11)	47 (41)	55 (47)	4.34
Leading an effective strategic planning process (n = 116)	0	3 (3)	12 (10)	48 (41)	53 (46)	4.30
Assessing the strengths, weaknesses, opportunities, and threats related to the pharmacy enterprise (n = 116)	0	5 (4)	6 (5)	60 (52)	45 (39)	4.25
Gaining buy-in from the C-suite (n = 116)	1 (1)	4 (3)	16 (14)	44 (38)	51 (44)	4.21
Creating a business plan (n = 115)	1 (1)	6 (5)	14 (12)	50 (43)	44 (38)	4.13

^a^Each item was rated with a 5-point Likert scale ranging from 1= “strongly disagree” to 5 = “strongly agree”

## Discussion

### Vibrancy and reach of pharmacy plans

**Figure 10.**
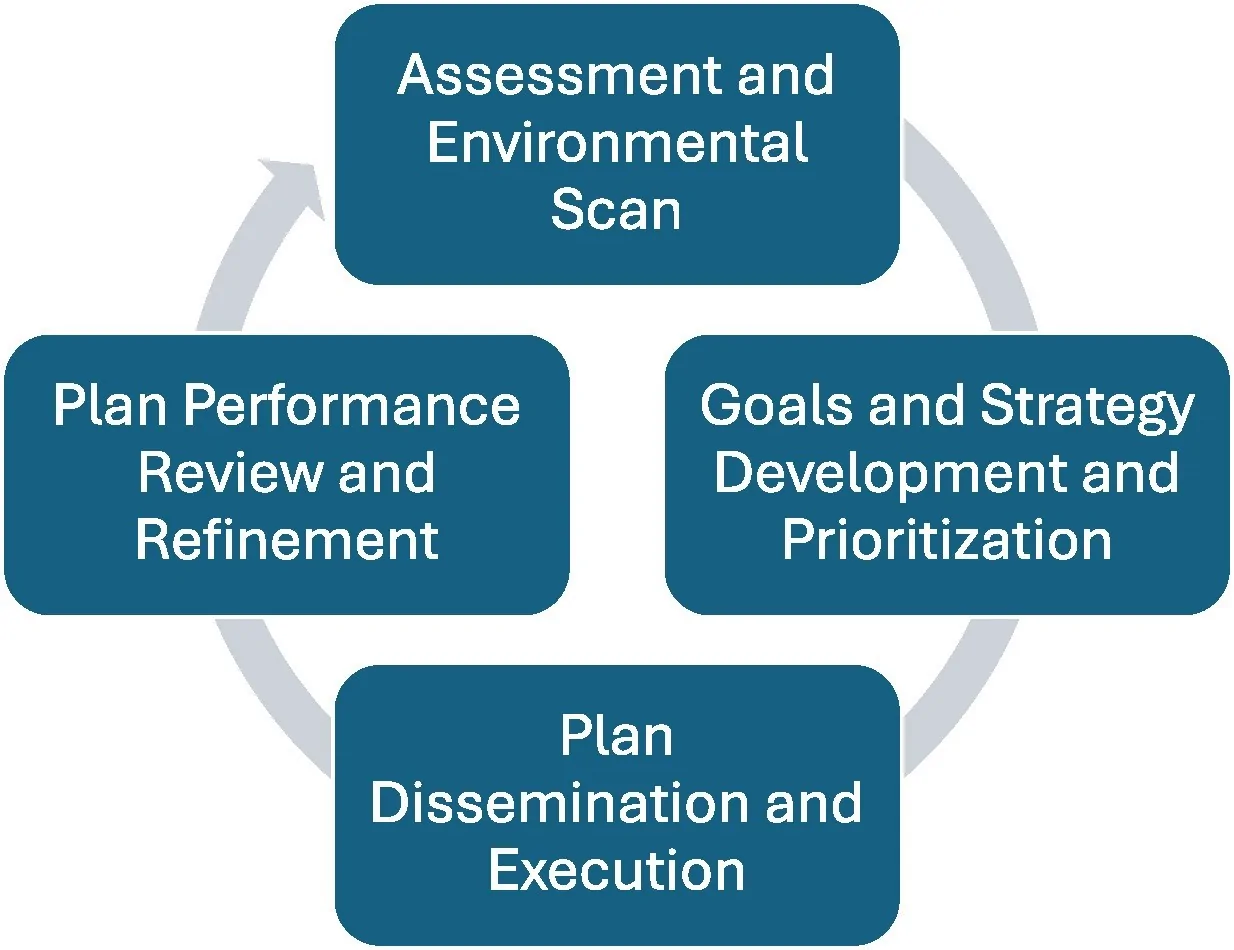
Four phases of health-system pharmacy strategic planning.

It is noteworthy that most survey respondents revisit their plans at least annually or quarterly. Frequent review of its strategic plan helps an enterprise remain forward-thinking, agile, and resilient throughout the 4 phases of strategic planning (Figure 10). This review requires an evaluation of current performance, understanding of environmental factors affecting health-system operations, and an evaluation of necessary adjustments to the plan to achieve enterprise strategic goals.

Most respondents indicated they have input into the overall organization’s strategic plan. This is vital for ensuring alignment across service line planning and a connection to the work of frontline teams. Many pharmacy leaders appear to be in a position to demonstrate how their enterprise can help the entire organization achieve its objectives. Future research could help identify how best to align plans across the organization as well as pharmacy service lines, particularly in health systems with multiple entities, and to better understand how the EWPSP integrates with service line–specific plans.

It is not surprising that the top issues addressed in the EWPSP were financial performance and ambulatory care services. This might reflect pharmacy alignment with the broader organization’s goals related to (1) revenue growth and operational excellence (for example, in infusion and specialty services) and (2) expanded primary and specialty care services, including support of alternative payment models.

### Process for developing the strategic plan

The relative strength of a strategic plan is influenced by the inputs from key stakeholders. Survey respondents indicated somewhat limited use of non-pharmacy resources in building the EWPSP. Pharmacy enterprise leaders should consider including in their planning process representatives from multidisciplinary teams and health-system leaders from outside of pharmacy and leaders from outside the enterprise to challenge internal biases. One way this can be done is to establish a “challenge network”—people who ask hard questions and challenge prevailing assumptions.^16^ They can (1) help identify potential pitfalls and barriers to success of the plan and (2) encourage openness to new ideas for addressing particular problems. Pharmacists and other healthcare experts with experience in different care settings or different organizations can also be helpful in a challenge network. Patients should be considered for engagement in the planning process, although that can be difficult to accomplish.

Most survey respondents indicated they use professional networking to conduct environmental scans. Health-system and pharmacy leaders should leverage professional networks (both internal and external) to identify opportunities, vet plans, and define metrics. Organizations should prioritize ways to support networking that foster strategic thinking.

### Strategic plan execution and monitoring

While establishing an EWPSP requires strategic *thinking*, execution of the plan requires strategic *management,* which should be a continual process to reassess progress and the need for plan revision.^17^ Most survey respondents reported having clear intradepartmental accountability for plan implementation and monitoring progress. However, the extent of external accountability was more variable. This may stem from lack of effective processes for including non-pharmacy and external stakeholders in developing the EWPSP or not having a structured method to communicate plan progress (eg, via committee or teams or individuals). Communicating the EWPSP outside of pharmacy is a way to (1) increase awareness of pharmacy’s value to the organization, (2) help coordinate initiatives across pharmacy and non-pharmacy teams, and (3) build support for allocating resources to major pharmacy initiatives. Synchronizing these conversations with the budgeting process is important.

Survey results indicated variability in how plan implementation is monitored. Slightly more than half of respondents “always” or “often” include strategic plan progress in their staff meetings or use their plan to establish performance objectives for team members. Pharmacy leaders should seek ways to engage teams in supporting strategic projects, which could foster team member job satisfaction and enterprise resilience.

The majority of respondents (54%) use a simple written report to share their EWPSP, while more than 40% of respondents use a planning tool to provide data visualization and ongoing monitoring. It would be useful to learn how those tools could be used to support plan implementation and monitoring, especially to share learnings across pharmacy enterprises.

### The value of strategic planning

Most survey respondents strongly agreed that (1) having a current strategic plan is critical to improving pharmacy operations and (2) execution of the plan helps advance the profession of pharmacy. Referring to the outcomes of their most recent planning cycle, a majority of respondents indicated that their plan “often” established new or expanded pharmacy services and that goals were “often” achieved within the designated timeframe. Because of the rapid pace of change today, those struggling with meeting timeline goals should consider allowing for adjustments as circumstances dictate; the traditional 3- to 5-year planning cycle may not be appropriate for many strategic initiatives.

Although this survey was not designed to examine the relationship between elements of strategic plan development and implementation outcomes, exploration of that connection in future research would be valuable. Findings in this area might help pharmacy leaders avoid the temptation to limit the focus of their planning to narrow pharmacy operational issues. Pharmacy practice leaders should spend substantial time on strategic thinking and then include in their EWPSP aspirational goals that bring greater value to the organization and the pharmacy profession.

### Strategic planning support

The ASHP/ASHP Foundation Pharmacy Forecast, which annually publishes strategic recommendations for pharmacy practice leaders, was the resource most frequently used by survey respondents in environmental scanning. Nearly equal in ranking was the professional networks of the respondents. This suggests an opportunity for professional organizations to consider how formal networking events (meetings and forums) could include elements of environmental scanning or offer strategic recommendations.

In counterbalance to the survey respondents’ apparent success in developing and implementing EWPSPs, they also had a high level of agreement that additional education, tools, and resources would help to improve their strategic planning efforts. Creating a resource center on certain topics, including example plans or guidance on resources for conducting an environmental scan, would help leaders build confidence in their strategic planning abilities. Furthermore, resources should address unique challenges and opportunities in a range of organizations. It would also be beneficial to formally incorporate competencies related to strategic planning in postgraduate year 2 health-system pharmacy administration and leadership residency training.

The highest-rated need for new planning resources was identification of emerging trends likely to have a future impact on the pharmacy enterprise. Pharmacy leaders must understand many aspects of healthcare, and it can be challenging to stay current in this regard. Creating ways to efficiently identify important issues and trends is critical to strategic planning given the rapidly changing and complex landscape of healthcare today.

### Limitations of the survey

The goal of this survey research was to document the current state of strategic planning within the pharmacy enterprises of nonfederal, nongovernment hospitals and health systems. The study was not designed to assess differences related to bed size or other institutional characteristics. While the final sample size fell within the targeted range based on statistical calculations, the overall response rate of 21% could indicate potential nonresponse bias. A higher percentage of respondents reported being part of a multihospital system than was reported in a national analysis (89% and 69%, respectively).^18^ Additionally, respondents were more likely to be members of ASHP (40%) than individuals in the entire invited sample (20%). Hence, it is not known to what extent the findings apply to all health-system pharmacy enterprises.

## Conclusion

The EWPSPs of the respondents typically span a timeframe of 3 to 5 years, and their plans are commonly revisited at shorter intervals. Plans of the respondents are developed and executed using a variety of resources and methods and are aligned with the overall goals of their organizations, though it was acknowledged there are opportunities to improve engagement of nonpharmacy stakeholders. Respondents indicated that having a strategic plan helped to expand existing services or to develop new services and helped to gain support from internal and external stakeholders. Respondents identified that new resources would be helpful in all areas of plan development and implementation, including conducting an environmental scan to determine risks and opportunities to ensure success of the pharmacy enterprise. There are opportunities for future research on strategic planning in health-system pharmacies, including examining how plans are aligned and integrated across service lines, which priorities are emphasized, and how specific planning elements influence outcomes.

## Data Availability

The data underlying this article will be shared upon reasonable request to the corresponding author.

## References

[1] Ivey MF. Rationale for having a chief pharmacy officer in a health care organization. Am J Health-Syst Pharm. 2005;62(9):975–978. doi:10.1093/ajhp/62.9.975 15851500

[2] Espinosa AM , Mehta A, Vlasimsky TB. ASHP statement on leadership as a professional obligation. Am J Health-Syst Pharm. 2025;82 (21):e921–e922. 40795358 10.1093/ajhp/zxaf180

[3] Amerine LB , Granko RP, Brummond PW, et al ASHP statement on the roles and responsibilities of the pharmacy executive. Am J Health-Syst Pharm. 2025;82 (23):e1020–e1022. 40627367 10.1093/ajhp/zxaf158

[4] Boyd AM , Clark JS, Kent SS. Strategic thinking in pharmacy. Am J Health-Syst Pharm. 2017;74(14):1103–1108. doi:10.2146/ajhp160356 28522644

[5] Rough S , Shane R, Armitstead JA, et al The high-value pharmacy enterprise framework: advancing pharmacy practice in health systems through a consensus-based, strategic approach. Am J Health-Syst Pharm. 2021;78(6):498–510. 33539506 10.1093/ajhp/zxaa431

[6] Birdwell SW , Pathak DS. Use of the strategic-planning process by hospital pharmacy directors. Am J Hosp Pharm. 1989;46(7):1361–1366. 2667353

[7] Harrison, DL. Strategic Planning by Institutional Pharmacy Administrators. Thesis. The University of Arizona; 1990. Accessed April 14, 2025. azu_td_1340287_sip1_m.pdf

[8] Small SM , Paratore PD, Dreyer JE, et al Successful implementation of a pediatric pharmacy team-level strategic plan during the COVID-19 pandemic. Am J Health-Syst Pharm. 2022;79(17):1411–1414. 35526276 10.1093/ajhp/zxac133 PMC9129157

[9] Kaczor C , Cole JW, Sapko MM, Kappeler KH. Realizing the vision for pediatric pharmacy practice advancement through strategic planning and implementation. Am J Health-Syst Pharm. 2020;77(6):466–473. 31960893 10.1093/ajhp/zxz340

[10] Shane R , Gouveia WA. Developing a strategic plan for quality in pharmacy practice. Am J Health-Syst Pharm. 2000;57(5):470–474. doi:10.1093/ajhp/57.5.470 10711529

[11] Eysenbach G. Improving the quality of Web surveys: the Checklist for Reporting Results of Internet E-Surveys (CHERRIES). J Med Internet Res. 2004;6(3):e34. doi:10.2196/jmir.6.3.e34. Published correction appears in *J Med Internet Res.* 2012;14(1):e8. doi:10.2196/jmir.2042 15471760 PMC1550605

[12] World Medical Association . World Medical Association Declaration of Helsinki: ethical principles for medical research involving human subjects. JAMA. 2013;310(20):2191–2194. doi:10.1001/jama.2013.281053 24141714

[13] IQVIA Hospital Database [propriety data]. IQVIA; 2023.

[14] Schneider PJ , Pedersen CA, Ganio MC, Scheckelhoff DJ. ASHP National Survey of Pharmacy Practice in Hospital Settings: Operations and Technology — 2023. Am J Health-Syst Pharm. 2024;81(16):684–705.

[15] Dillman DA , Smyth JD, Christian LM. Internet, Phone, Mail, and Mixed-Mode Surveys: The Tailored Design Method. 4th ed. Wiley; 2014.

[16] Grant A. Think Again: The Power of Knowing What You Don’t Know. Audiobook. Penguin Audio; 2021.

[17] Adobe. Strategic planning and management. Adobe for Business blog. Published July 16, 2025. Accessed August 15, 2025. https://business.adobe.com/blog/basics/strategic-planning

[18] Levinson Z , Hulver S, Godwin J, et al Key facts about hospitals. KFF. Published February 19, 2025. Accessed August 15, 2025. https://www.kff.org/key-facts-about-hospitals/?entry=the-hospital-industry-number-of-hospitals

